# Purpose in Life: A Reconceptualization for Very Late Life

**DOI:** 10.1093/geroni/igab046.981

**Published:** 2021-12-17

**Authors:** Keith Anderson, Noelle Fields, Jessica Cassidy, Lisa Peters-Beumer

**Affiliations:** 1 University of Texas at Arlington, Arlington, Texas, United States; 2 Concordia University Chicago, River Forest, Illinois, United States

## Abstract

Across disciplines, we have long sought to understand the factors that contribute to purpose in life. Theorists have posited that having life goals, feeling productive, and remaining active are essential contributing elements to purpose in life (Crumbaugh & Maholick, 1969; Rowe & Kahn, 1997; Ryff, 1989). While these factors can undoubtedly contribute to purpose in life, they may not fully explain purpose in life for older adults in very late life (85 years old and older) who have long past and short future time horizons. In this presentation, we explore the concept of purpose in life for older adults in very late life and how current measures may not fully or accurately apply to this group. We examine the two most commonly used measures of purpose in life, the Purpose in Life Test (Crumbaugh & Maholick, 1964, 1969) and the Ryff Purpose Subscale (Ryff, 1989; Ryff & Keyes, 1995), and identify specific items that should be reconsidered for use with older adults in very late life. We then reconceptualize purpose in life for the oldest old based on several foundational theories, including Socioemotional Selectivity Theory, the Theory of Gerotranscendence, and Terror Management Theory. Stemming from this analysis, we posit that purpose in life in very life consists of three domains – the very long past, the very near future, and the transcendental post-mortem. Based upon this reconceptualization, we recommend the development of new measures of purpose of life in very late life that capture these three domains.

